# Rheb may complex with RASSF1A to coordinate Hippo and TOR signaling

**DOI:** 10.18632/oncotarget.8447

**Published:** 2016-03-28

**Authors:** Nicholas Nelson, Geoffrey J. Clark

**Affiliations:** ^1^ Department of Biochemistry and Molecular Biology, University of Louisville, Louisville, KY, USA; ^2^ Department of Pharmacology and Toxicology, University of Louisville, Louisville, KY, USA

**Keywords:** Ras, Rheb, Hippo, TOR, RASSF1A

## Abstract

The TOR pathway is a vital component of cellular homeostasis that controls the synthesis of proteins, nucleic acids and lipids. Its core is the TOR kinase. Activation of the TOR pathway suppresses autophagy, which plays a vital but complex role in tumorigenesis. The TOR pathway is regulated by activation of the Ras-related protein Rheb, which can bind mTOR. The Hippo pathway is a major growth control module that regulates cell growth, differentiation and apoptosis. Its core consists of an MST/LATS kinase cascade that can be activated by the RASSF1A tumor suppressor. The TOR and Hippo pathways may be coordinately regulated to promote cellular homeostasis. However, the links between the pathways remain only partially understood. We now demonstrate that in addition to mTOR regulation, Rheb also impacts the Hippo pathway by forming a complex with RASSF1A. Using stable clones of two human lung tumor cell lines (NCI-H1792 and NCI-H1299) with shRNA-mediated silencing or ectopic overexpression of RASSF1A, we show that activated Rheb stimulates the Hippo pathway, but is suppressed in its ability to stimulate the TOR pathway. Moreover, by selectively labeling autophagic vacuoles we show that RASSF1A inhibits the ability of Rheb to suppress autophagy and enhance cell growth. Thus, we identify a new connection that impacts coordination of Hippo and TOR signaling. As RASSF1A expression is frequently lost in human tumors, the RASSF1A status of a tumor may impact not just its Hippo pathway status, but also its TOR pathway status.

## BACKGROUND

Rheb is a Ras-related small GTPase which is broadly expressed in human tissue [1][2]. It is negatively regulated by the TSC1/TSC2 tumor suppressor complex, which exhibits GAP activity against Rheb [3-5]. Mutations in the TSC complex are found in human tumors [6]. Moreover, hereditary defects in the TSC complex lead to Tuberous Sclerosis, a genetic disease characterized by constitutively active Rheb and a predisposition to CNS and Renal neoplasms [7-9]. In experimental systems, Rheb can promote the transformed phenotype [10]. However, it can also induce apoptosis [11, 12]. This suggests that the action of Rheb in a cell may be context dependent [13].

Activated Rheb binds and activates the mTOR kinase to constitutively drive the TOR pathway [3, 14]. The TOR pathway plays a key role in cellular homeostasis [15, 16]. The mTOR kinase phosphorylates the S6 kinases (S6K1 and S6K2) and the eukaryotic translation initiation factor 4E (4E-BP1), which serve to control protein synthesis [17]. mTOR also regulates nucleic acid and lipid biosynthesis [16, 18]. One of the major effects of the activation of mTOR by Rheb is to suppress autophagy [19].

Autophagy is a cellular catabolic degradation response to stress or starvation where the cell's proteins and organelles are digested and recycled to sustain cellular metabolism [20][21]. Autophagy clearly has a pro-survival role, and has been implicated in the persistence of minimal residual disease in cancer [20, 22]. Moreover, defects in the autophagic processes *in vivo* can inhibit the tumorigenicity of oncogenic Ras [23]. However, it can also lead to cell death [24, 25], and defects in autophagic components can lead to tumor development in transgenic mice. Thus, the role of autophagy in cancer is complex and appears somewhat contradictory [26][27].

In addition to autophagy, Rheb may also modulate apoptosis [11, 12]. This should not be surprising, as there are clearly links between autophagy and apoptotic death [28]. It has been suggested that the action of Rheb on apoptosis may be due, in part, to an interaction with the potential mTOR inhibitor FKBP38 [12]. This interaction serves to suppress apoptosis by releasing the anti-apoptotic proteins Bcl2 and Bcl-XL from an inactive complex. Therefore, much like its cousin Ras, Rheb appears to act by binding and activating multiple effector proteins to establish a complex signaling network [13].

The Hippo pathway is plays a critical role in many aspects of cellular growth, development and death [29]. It was first identified as a regulator of organ size [30]. At its core lies an MST/LATs kinase cascade terminating in the phosphorylation of the transcriptional co-regulators YAP and TAZ. Phosphorylation of YAP and TAZ by the Hippo pathway leads to their degradation and has a pro-apoptotic effect.

As both the TOR and the Hippo pathways are involved in control of organ size, it might be anticipated that the pathways should exhibit co-regulation and cross talk. Indeed, several points of interaction between the pathways have now been identified [31][32][33]. Moreover, both pathways can be activated by the Ras oncoprotein [29, 32]. Ras activates TOR *via* the PI-3 kinase pathway [34, 35] and Hippo *via* RASSF1A [36].

RASSF1A is a pro-apoptotic tumor suppressor that contains a Ras Association domain, and can bind Ras and certain Ras related proteins [37]. RASSF1A binds the MST kinases, which form the start of the canonical Hippo pathway [38]. The binding of activated K-Ras to RASSF1A results in activation of MST1 and hence the Hippo pathway, leading to apoptosis [39, 40].

We now identify an additional level of co-regulation between Hippo and TOR. We show that Rheb can complex not only with TOR, but also with the Hippo pathway regulating tumor suppressor RASSF1A. Thus, when Rheb is constitutively activated by defects in the TSC GAP complex, in addition to a direct activation of mTOR, Rheb may also bind and modulate RASSF1A/Hippo at the same time. We find that in the presence of RASSF1A, Rheb stimulates the Hippo pathway, but is suppressed in its ability to activate the TOR pathway. Consequently, RASSF1A enhances autophagy and blocks the ability of Rheb to suppress autophagy. Thus, RASSF1A levels in a cell may have a profound effect on the net biological effect of Rheb activation.

## RESULTS

### Rheb interacts with RASSF1A

**Figure 1:**
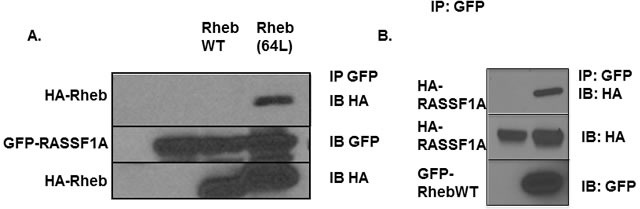
Rheb can complex with RASSF1A **A.** HEK-293T cells were transiently transfected with HA-tagged wild type Rheb, Rheb(64L) and GFP-tagged RASSF1A expression constructs. Twenty four hours after transfection the cells were lysed and equal amounts of protein were immunoprecipitated (IP) for GFP. The immunoprecipitate was fractionated on an SDS-polyacrylamide gel and then immunoblotted (IB) with anti-HA and anti-GFP antibodies. **B.** HEK-293T cells were transiently transfected with HA-tagged wild-type Rheb and GFP-tagged RASSF1A expression constructs. Twenty four hours after transfection the cells were lysed and equal amounts of protein were immunoprecipitated (IP) for GFP. The immunoprecipitate was fractionated on an SDS-polyacrylamide gel and then immunoblotted (IB) with anti-HA and anti-GFP antibodies.

The tumor suppressor RASSF1A can bind K-Ras and certain Ras related proteins [37, 41]. Since Rheb shows considerable sequence homology with the Ras core effector domain, and functional and physical interactions between Rheb and other Ras effectors have been shown previously [11, 42], we sought to determine whether Rheb could form a complex with RASSF1A in human cells. We used exogenous expression experiments to show that an activated form of Rheb, co-precipitated with RASSF1A when the proteins were co-expressed in HEK-293T cells. The activated mutant form exhibited preferential binding compared to the wild type form (Figure 1A). In Figure 1A we see no apparent interaction with the wild type form of Rheb. However, we could detect wild type Rheb in complex with RASSF1A, but the levels were so much weaker than the activated form it was difficult to observe both on the same blot as the activated band became over-exposed. Therefore, we show wild type Rheb alone complexing with RASSF1A in Figure 1B. This demonstrates that the proteins can interact, and the interaction is dependent upon the activation state of Rheb (Figure 1A). We have also performed experiments designed to detect the presence of a stable, endogenous complex of the two proteins. However, we have been unable to obtain convincing, publication quality results.

### Rheb activates hippo *via* RASSF1A

**Figure 2:**
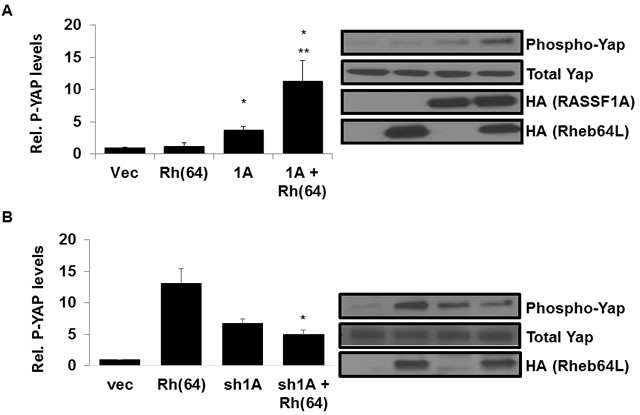
Rheb activates Hippo *via* RASSF1A **A.** Rheb and RASSF1A synergize to phosphorylate YAP. (Left panel) NCI-H1299 cells stably transfected with vectors expressing HA-tagged RASSF1A and HA-tagged Rheb(64L) were seeded to confluency and serum starved overnight, and equal amounts of protein lysates were analyzed by Western blotting for phosphorylated Yap expression using anti-phospho-Yap antibodies. Immunoreactive bands were quantified by densitometry and the results plotted as a bar graph showing relative phospho-YAP levels normalized to vector transfected cells. Values that are significantly different are indicated by an asterisk as follows: *, *P* < 0.05 compared to the value for vector control cells. **, *P* < 0.05 compared to the value for cells expressing RASSF1A. (Right panel) Expression levels of each protein are shown in a Western blot. Exogenous protein expression was detected using anti-HA antibodies. The Western blot shown is representative of three independent experiments. **B.** RASSF1A loss impairs YAP phosphorylation by Rheb. NCI-H1792 cells stably transfected with vectors expressing shRNA-RASSF1A and HA-tagged Rheb(64L) were seeded to confluency and serum starved overnight, and equal amounts of protein lysates were analyzed by Western blotting for phosphorylated Yap expression using anti-Phospho-Yap antibodies. Immunoreactive bands were quantified by densitometry and the results plotted as a bar graph showing relative phospho-YAP levels normalized to vector transfected cells. Values that are significantly different are indicated by an asterisk as follows: *, *P* < 0.05 compared to the value for cells expressing Rheb(64L) (right). Expression levels of each protein are shown in a Western blot. Exogenous protein expression was detected using anti-HA antibodies. The Western blots shown are representative of three independent experiments.

RASSF1A activates the Hippo pathway by directly binding and activating MST kinases, ultimately promoting phosphorylation and inactivation of transcriptional regulator YAP and driving apoptosis [43]. To determine whether Rheb has an effect on Hippo signaling by RASSF1A, we stably introduced activated Rheb(64L) into our previously established H1299 +/− RASSF1A matched pair cell lines described in [44]. Briefly, expression vectors were used to generate H1299 cell lines (negative for endogenous RASSF1A expression) that stably re-express exogenous RASSF1A. We then stably transfected the H1299 cells +/− for RASSF1A with Rheb(64L) and examined the cell lysates for YAP phosphorylation. H1299 cells with restored RASSF1A expression showed enhanced YAP phosphorylation in the presence of activated Rheb compared to cells expressing exogenous RASSF1A or Rheb alone (Figure 2A).

To confirm the Rheb/RASSF1A effect on Hippo signaling, we used our previously validated NCI-H1792 RASSF1A knockdown matched pair of cell lines [44] to generate a matched set that stably expressed Rheb(64L). Having stably transfected the RASSF1A +/− matched pair cells with activated Rheb(64L) we measured the levels of YAP phosphorylation in the cell lysates. Cells where expression of endogenous RASSF1A is inhibited by an shRNA construct show a decrease in YAP phosphorylation in the presence of activated Rheb compared to cells that retain endogenous RASSF1A expression (Figure 2B).

Thus, when we restore RASSF1A expression to H1299 cells we induce the ability of Rheb to activate the Hippo pathway and when we remove RASSF1A from H1792 cells the ability of Rheb to activate Hippo is lost.

### Rheb activation of TOR is suppressed by RASSF1A

**Figure 3:**
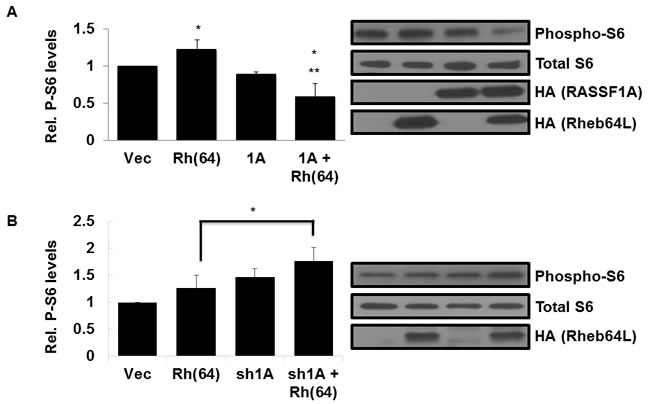
Rheb activation of TOR is suppressed by RASSF1A **A.** RASSF1A suppresses TOR activation by Rheb. (Left panel) NCI-H1299 cells described in Figure 2A were Western blotted for phosphorylated S6 expression using anti-Phospho-S6 antibodies. Immunoreactive bands were quantified by densitometry and the results plotted as a bar graph showing levels of phospho-S6 normalized to vector transfected cells. Values that are significantly different are indicated by an asterisk as follows: *, *P* < 0.05 compared to the value for vector control cells. **, *P* < 0.01 compared to the value for cells expressing Rheb(64L). (Right panel) Expression levels of each protein are shown in a Western blot. Exogenous protein expression was detected using anti-HA antibodies. The Western blot shown is representative of three independent experiments. **B.** Loss of RASSF1A restores S6 phosphorylation by Rheb. (Left panel) NCI-H1792 cells described in Figure 2B were Western blotted for phosphorylated S6 expression using anti-Phospho-S6 antibodies and immunoreactive bands were quantified by densitometry and the results plotted as a bar graph showing levels of phospho-S6 normalized to vector transfected cells. Values that are significantly different are indicated by an asterisk as follows: *, *P* < 0.05 compared to the value for cells expressing Rheb(64L). (Right panel) Expression levels of each protein are shown in a Western blot. Exogenous protein expression was detected using anti-HA antibodies. The Western blot shown is representative of three independent experiments.

Activated Rheb binds and activates the mTOR kinase, constitutively driving the TOR pathway [3, 14]. To determine whether RASSF1A has an effect on Rheb activation of TOR, we measured the levels of mTOR-target S6 kinase activation in lysates from our stable H1299 +/− RASSF1A, +/− Rheb(64L) matched set cell system described above. H1299 cells with activated Rheb and restored RASSF1A expression showed a significant decrease in S6 phosphorylation compared to cells expressing Rheb(64L) alone (Figure 3A), suggesting that the interaction of RASSF1A with Rheb diverts it from activating TOR.

To confirm that Rheb/TOR signaling suppression is occurring *via* RASSF1A, we examined lysates from our NCI-H1792 RASSF1A +/−, Rheb +/− matched set cell system described above for levels of S6 phosphorylation. As expected, cells where expression of endogenous RASSF1A is inhibited by an shRNA construct exhibit enhanced S6 phosphorylation by activated Rheb (Figure 3B).

Thus, when we restore RASSF1A expression to H1299 cells we inhibit the ability of Rheb to activate TOR and when we remove RASSF1A from H1792 cells we enhance the ability of Rheb to activate TOR.

### Rheb-mediated growth enhancement is suppressed by RASSF1A

**Figure 4:**
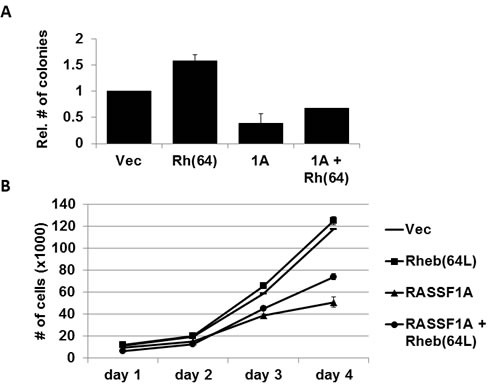
Rheb-mediated growth enhancement is suppressed by RASSF1A **A.** RASSF1A impairs Rheb-mediated anchorage-independent growth. NCI-H1299 cells stably transfected with vectors expressing HA-tagged RASSF1A and HA-tagged Rheb(64L) were plated in soft agar. Frequency of colony formation was quantified and data was plotted as a bar graph showing the relative number of colonies in each normalized to vector control cells. Experiments were done twice in duplicate and the error bars show SD. **B.** RASSF1A suppresses Rheb-mediated growth enhancement. Standard growth curves were performed on matched sets of H1299 cells described in **A.** in 2-dimensional cell culture. Experiments were done in duplicate and the error bars show SD.

Overexpression of activated Rheb has been shown to enhance anchorage-independent growth of cells in soft agar [10]. To determine whether the tumor suppressor RASSF1A has an effect on Rheb-mediated colony formation of human tumor cells, we grew cells from our matched set of H1299 +/− RASSF1A +/− Rheb(64L) stable transfectants in soft agar for two weeks. RASSF1A dramatically reduced the anchorage-independent growth of these cells induced by activated Rheb (Figure 4A). Standard growth analysis performed on the same matched set of H1299 cells over a four day period exhibited similar growth inhibitory results (Figure 4B). Thus, RASSF1A appears to be suppressing Rheb-mediated growth and transformation.

### RASSF1A blocks Rheb suppression of autophagy

**Figure 5:**
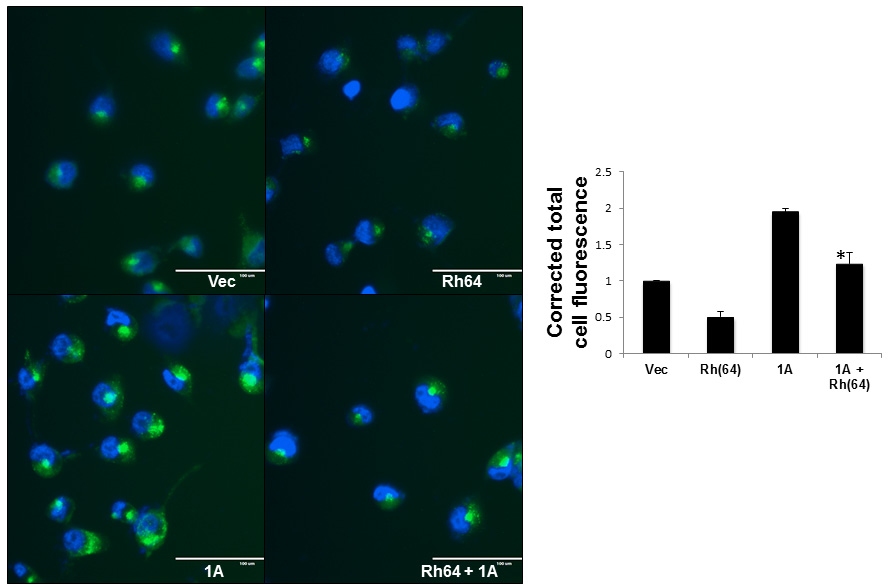
RASSF1A blocks Rheb suppression of autophagy The NCI-H1299 cell matched set described in Figure 2 were grown on 35mm dishes to ~60% confluency and nuclei were stained with Cyto-ID detection dye and Hoechst 33342 stain. The stained cells were observed under fluorescent microscope. Scale bar indicates 100μm. (right) Corrected total cell fluorescence (CTCF) of a minimum of 50 cells per treatment was calculated using imageJ software, and the results of three independent experiments plotted as a bar graph. Data is expressed as corrected total cell fluorescence [CTCF = Integrated density - (Area of total cell x mean fluorescence of background)] normalized to vector control cells. Values that are significantly different are indicated by an asterisk as follows: *, *P* < 0.05 compared to the value for Rh(64) cells.

Rheb activates mTOR to suppress autophagy and promote transformation [19]. Here, we see RASSF1A interfering with the ability of Rheb to stimulate the TOR pathway. Thus, we wondered what effect RASSF1A expression might have on autophagy suppression by Rheb. We performed autophagy assays on the stable NCI-H1299 matched set cell system using a Cyto-ID Autophagy Detection Kit (Enzo, Farmingdale NY). Figure 5 shows the result of three separate assays. In each case, we see Rheb suppressing autophagy in the absence of RASSF1A, as expected. However, in the RASSF1A expressing cells, autophagy was elevated approximately twofold and RASSF1A acted to impair the ability of Rheb to suppress autophagy.

## DISCUSSION

The mTOR kinase plays a key role in cellular homeostasis [45]. mTOR kinase forms two functional complexes TORC1 and TORC2. TORC1 is the best characterized and regulates cell growth and protein synthesis by modulating enzymes involved in protein translation [46]. It is also phosphorylates and regulates essential components of the autophagic process. mTOR in the TORC1 but not the TORC2 complex can be regulated by Rheb [47, 48]

The Hippo pathway is a major cellular signaling pathway that can regulate cell growth, differentiation and death [49]. At its core lies a kinase cascade where the kinases MST1 and 2 phosphorylate and activate the kinases LATS1 and 2. The LATs kinases then phosphorylate the transcriptional co-activators YAP and TAZ, promoting their degradation.

Aberrant activation of the TOR pathway is found in many tumors and is the major component of the genetic disease Tuberous sclerosis [50]. Here, mTOR is constitutively activated due to loss of function of the TSC complex, which normally acts as a GAP, or negative regulator of the Ras related protein Rheb. Active Rheb binds mTOR promoting its activation. Deregulation of Hippo pathway components can also be detected in human cancer [51]. This can lead to stabilization of the transcriptional co-activators YAP and TAZ, which are oncogenic.

As both pathways play an essential role in the regulation of organ size [31], it is to be expected that their regulation can be coordinated. One level of co-regulation may be *via* the Ras oncoprotein. Ras can stimulate the TOR pathway *via* a PI-3 kinase /AKT signaling route [34, 35]. Ras also activates the Hippo pathway *via* the RASSF1A tumor suppressor [40, 52]. RASSF1A binds MST kinases and its interaction with Ras promotes activation of the Hippo kinase cascade. RASSF1A is frequently down-regulated in human tumors, thus uncoupling Ras from Hippo. Activation of an apoptotic signaling pathway by an oncogene may seem counter-intuitive. However, although powerfully transforming, the Ras oncoprotein can also stimulate apoptosis and senescence [53, 54]. These paradoxical effects may be part of an evolutionary fail-safe mechanism used by multi-cellular organisms to remove dysfunctional cells from the system, preventing their development into tumors.

Here, we identify a further level of co-regulation of Hippo/TOR by the Ras related protein Rheb. Rheb is known to bind and activate mTOR [3, 14] but it also has the potential to bind to some of the same effectors as Ras [42]. We show that Rheb interacts not just with mTOR, but also with the upstream Hippo regulator RASSF1A. This allows Rheb to stimulate the Hippo pathway. We have been unable to confirm an endogenous interaction between RASSF1A and wild type Rheb at this point. The immune-reagents are not optimal for these proteins but we suspect this reflects a relatively transient interaction between the wild type proteins. However, the activated form of Rheb bound much better than the wild type. As Rheb is mutated recurrently in tumors [55], it may be that this interaction is most pertinent in activated Rheb tumor cells.

The Hippo pathway can cross regulate the TOR pathway, as YAP expression up-regulates miRNA29 whose target is the phosphatase PTEN [33]. Down-regulation of PTEN serves to activate the PI-3 kinase pathway, and thereby, mTOR. Thus, stimulation of Hippo by Rheb, leading to YAP degradation should block this action and inhibit the TOR pathway. Alternatively, RASSF1A could also be competing with mTOR for binding to Rheb.

RASSF1A is the most frequently inactivated tumor suppressor in human cancer [37]. It exhibits aberrant promoter methylation and silencing in almost 50% of tumors. Based on our data, we would expect cells that have lost RASSF1A expression to show decreased YAP phosphorylation and therefore enhanced mTOR activity in the presence of activated Rheb. Indeed, when we knocked down RASSF1A in NCI-H1792 human lung cancer cells we saw a decrease in Hippo pathway activation and an increase in TOR pathway activation in the presence of activated Rheb (Figure 3A-3B). Based on these results, it would be interesting to see if tumors from patients with TSC disease that have also lost RASSF1A expression exhibit worse prognosis due to deregulation of the Rheb/RASSF1A signaling pathway and hyperactivation of mTOR. The role of RASSF1A in TSC provoked tumors has not been investigated.

Since constitutively active Rheb has been shown to induce oncogenic transformation in cell culture that is dependent upon mTOR activity [10], we expected inhibition of mTOR by RASSF1A to suppress oncogenic growth and transformation by activated Rheb. This turned out to be the case. When we examined the role of the Rheb/RASSF1A interaction on apoptosis, we were unable to detect significant changes in apoptosis induction (data not shown). Thus, we examined the effects on the interaction on autophagy.

The role of autophagy in tumorigenesis appears complex and somewhat contradictory [26]. There is a clear tumor suppressor role for autophagy. Many of the proteins that negatively regulate mTOR are tumor suppressors, such as PTEN, AMPK, and TSC1/2 [56]. Meanwhile, proteins that activate TOR and inhibit autophagy tend to be oncoproteins, such as PI3K, Ras, RHEB, and AKT [56][57]. Our findings add RASSF1A to the group that can promote autophagy, and this may play an unexpected role in its tumor suppressive properties. It may be particularly interesting that RASSF proteins can support autophagy bearing in mind their recent connection with Oncogene induced senescence (OIS) [58, 59]. Autophagy appears to play a role in the development of OIS [60, 61], which is thought to be a major barrier to malignancy [62].

However, although considerable evidence supports a role for autophagy in tumor suppression, Ras mediated malignant transformation appears to require at least some autophagic capacity [63, 64]. *In vivo* studies have shown that the key elements of the autophagic process are essential for the induction of K-Ras dependent lung tumors [23]. Autophagy has also been strongly implicated as a mechanism whereby tumor cells can survive in a dormant state to induce minimal residual disease [20, 22]. It is interesting to note that in transgenic studies, RASSF1A homozygous knockout mice develop fewer spontaneous tumors than the RASSF1A heterozygous knockout mice [65]. Moreover, RASSF1A is seldom completely deleted in human tumors, rather transcriptionally repressed by epigenetic mechanisms [37]. This suggests that retaining some low level of RASSF1A expression may be important for transformation. Perhaps a required role in supporting a basal autophagic capacity could provide a mechanism to explain these observations.

Thus, we identify a novel interaction between Rheb and the tumor suppressor RASSF1A. This interaction may allow coordinate upregulation of Hippo while TOR is suppressed to modulate the balance of apoptosis and autophagy.

## MATERIALS AND METHODS

### Plasmids and DNA

Expression plasmids for RASSF1A, wild type Rheb and constitutively-activated Rheb(64L) have been described previously [66, 67].

### Tissue culture and cell lines

Cells were obtained from the ATCC (Manassas, VA). NCI-H1299 and NCI-H1792 cells were cultured in RPMI 1640 medium supplemented with 10% FBS (Valley Biologicals, VA). The NCI-H1299 matched set and the NCI-H1792 matched set of RASSF1A knockdown cells have been described previously [44]. HEK-293T cells were cultured in DMEM with 10% FBS. Stable transfectants were generated by transfecting cells each with 1μg of plasmid DNA using jetPRIME (PolyPlus, Strasbourg, Fr) transfection reagent following the manufacturer's instructions. RASSF1A transfectants were selected in 500μg/mL G418 (Invitrogen, Carlsbad CA). Rheb transfectants were selected in 75g/mL Hygromycin B (Life Technologies, Grand Island NY). Transient transfections were performed using jetPRIME and 1μg of each plasmid DNA. Growth assays were performed by plating H1299 cells at 4×10^4^ cells per 60-mm dish. Anchorage-independent growth was determined by colony formation in soft agar. Matched sets of H1299 (2×10^3^ cells/well) cells were mixed were mixed with 2mL of culture media containing 0.3% agar (Difco, Franklin Lakes, NJ) and then overlaid on 1mL of 0.6% agar in 12 well plates. Colonies were counted after two weeks of incubation at 37C.

### Antibodies

Anti -GFP antibodies (# 9996) were obtained from Santa Cruz Biotechnology Inc. (Santa Cruz, CA). The HA antibody was obtained from Sigma (Newark, NJ). S6, Phospho-S6, YAP and Phospho-YAP antibodies (#2212, #2211, #4912, #4911) were obtained from Cell Signaling (Danvers, MA) Trueblot secondary antibodies were purchased from eBioscience (San Diego, CA).HRP conjugated or Trueblot secondary antibodies were purchased from eBioscience (San Diego, CA).

### Western analysis and immunoprecipitation

For signaling assays, NCI-H1299 and NCI-H1792 cells were seeded at confluence and serum starved overnight. Cells were lysed in RIPA buffer (Sigma) and the lysates Western blotted on a 4-15% Tris-glycine gel and transferred to a 0.2μm nitrocellulose using a Mini-PROTEAN electrophoresis system (Bio-Rad Laboratories, Hercules, CA). For co-immunoprecipitation, HEK 293T cells were transfected as above and lysed in modified RIPA buffer (150mM NaCl, 50mM Tris, pH 7.5, 1% NP-40). Precleared lysates were immunoprecipitated with GFP-Trap agarose beads (Allele Biotech, San Diego CA) or primary antibody as appropriate and washed with lysis buffer. Western blots were developed using a Pierce ECL detection system (Thermo Scientific, Rockford IL) and autoradiography film (MidSci, St. Louis, MO).

### Autophagy assays

Autophagy assays were performed using a Cyto-ID Autophagy Detection Kit (Enzo Life Sciences, Farmingdale, NY) using the protocol described by the manufacturer. Live cells were analyzed by fluorescent microscopy using an EVOS FL imaging system (Life Technologies). Corrected total cell fluorescence was calculated as described in [68] using ImageJ software.

### Image acquisition and processing

Images were scanned and quantified using a Pharos FX plus Molecular Imager (Bio-Rad) and Quantity One software (Bio-Rad).
